# Asthma's effect on brain connectivity and cognitive decline

**DOI:** 10.3389/fneur.2022.1065942

**Published:** 2023-02-03

**Authors:** Tao Wang, Xin Huang, Jun Wang

**Affiliations:** ^1^Medical College of Nanchang University, Nanchang, China; ^2^The Second Department of Respiratory Disease, Jiangxi Provincial People's Hospital, The First Affiliated Hospital of Nanchang Medical College, Nanchang, China; ^3^Department of Ophthalmology, Jiangxi Provincial People's Hospital, The First Affiliated Hospital of Nanchang Medical College, Nanchang, China

**Keywords:** asthma, dVMHC, fMRI, brain activity, cognition function

## Abstract

**Objective:**

To investigate the changes in dynamic voxel mirror homotopy connection (dVMHC) between cerebral hemispheres in patients with asthma.

**Methods:**

Our study was designed using a case-control method. A total of 31 subjects with BA and 31 healthy subjects with matching basic information were examined using rsfMRI. We also calculated and obtained the dVMHC value between the cerebral cortexes.

**Results:**

Compared with the normal control group, the dVMHC of the lingual gyrus (Ling) and the calcarine sulcus (CAL), which represented the visual network (VN), increased significantly in the asthma group, while the dVMHC of the medial superior frontal gyrus (MSFG), the anterior/middle/posterior cingulate gyrus (A/M/PCG), and the supplementary motor area (SMA) of the sensorimotor network decreased significantly in the asthma group.

**Conclusion:**

This study showed that the ability of emotion regulation and the efficiency of visual and cognitive information processing in patients with BA was lower than in those in the HC group. The dVMHC analysis can be used to sensitively evaluate oxygen saturation, visual function changes, and attention bias caused by emotional disorders in patients with asthma, as well as to predict airway hyperresponsiveness, inflammatory progression, and dyspnea.

## Introduction

Asthma is a widespread and common condition among the population in developing countries, causing poor health for more than 300 million people (1, 2). The clinical signs of asthma are characterized by dynamic changes in each stage and heterogeneity at the beginning of the illness (2, 3). Insufficient oxygen supply to the cerebrum of patients with asthma may lead to structural changes (4). Asthma keeps the brain deprived of oxygen, which can cause brain cell damage and ischemic demyelination of white matter (5, 6). Chronic intermittent hypoxia may result in DNA breakage, ATP depletion, mitochondrial dysfunction, and free radical production that reduce hippocampal neuron dendrite length and synaptic proteins (7). VonLeupoldt et al. (8) found that insular cortex activity decreased and PAG activity increased in patients with asthma, which may represent the neuronal mechanism of habitual reduction of unpleasant emotions caused by dyspnea in patients with asthma (9). In addition, patients with asthma are more likely to be affected by emotion and are under chronic stress. Some studies found that chronic stress may play an important role in delayed anaphylaxis by activating insular and ACC (10, 11). The risk of anxiety and depression in patients with asthma is two times that of healthy people (12). This may be related to the decreased biological activity of tryptophan hydroxylase (13). Wang et al. (14) found that the volume of gray matter in the right superior temporal gyrus, the right middle frontal gyrus, and the bilateral anterior cuneate lobe in patients with asthma and depression was lower than that in patients with simple asthma. Zhang et al. (15) found that the functional connection between the left ventral forebrain island and the left middle temporal gyrus in asthma patients with depression was stronger than in asthma patients without depression and healthy controls. Glucocorticoids are one of the main treatments for patients with asthma. Brown et al. (16, 17) found that the amygdala and hippocampus volumes decreased in patients who received long-term glucocorticoid treatment. The degree of decrease was closely related to the duration of glucocorticoid treatment.

Functional changes in the cerebrum area can also affect the course of the disease. For example, under the influence of negative stimuli, the anterior cingulate gyrus(ACC) and the limbic lobe of patients with asthma directly or indirectly promote the release of stress hormones by releasing neuropeptides, thus amplifying Th2 inflammation of the respiratory tract (18, 19). A previous rsfMRI study found that both the regional activity of the default mode network and the functional connectivity of the sensorimotor and visual networks were abnormal in patients with asthma, as determined through the calculation of local brain activity (ALFF) and neural network function [degree centrality (DC) and functional connectivity (FC)]. These independent changes confirm that the changes in the gyrus DC are related to the degree of airway obstruction (20). The change in the sensorimotor network is related to respiratory amplitude in patients with asthma (21). Li et al. also found changes in DC values in different brain regions of patients with asthma. The difference is that, in addition to the above areas, there are abnormal functional connections in the cortex-basal ganglia and the frontal-parietal networks in patients with asthma (22). In addition, Zhang et al. (15) found that the FC between the left vAI and the bilateral parietal lobe decreased, while the parietal lobe was responsible for the perception and processing of signals related to dyspnea (23). At the same time, the FC between vAI and ACC on the left increased, and the stronger the ACC activity, the more serious the airway inflammation (24). This study also found an increase in FC between left vAI and left MTG in patients with depressive asthma, while increased MTG response was associated with mood disorders (25).

It has been confirmed that cognitive impairment exists both in grown-ups and youngsters with asthma (26, 27). Such cognitive impairment in patients with asthma is thought to be caused by changes in the brain's structure. Through a retrospective analysis, Carlson et al. (28) found that patients with asthma have hippocampal volume atrophy, which is positively correlated with cognitive impairment. Low levels of hippocampal NAA (an indicator of neuronal density and integrity of N-acetyl aspartate) in patients with asthma can lead to poor performance of declarative memory tasks (28). In addition, chronic intermittent hypoxia causes damage to the hippocampus, which can lead to decreased spatial learning, affecting cognitive function (7). Repeated asthma attacks and persistent poor control can lead to sleep disorders and affect the cognitive function of the patient's brain (29). However, the exact neural mechanism of cognitive impairment in asthmatic patients remains unclear.

Previous studies revealed the changes and significance of functional connections in resting states in specific brain regions related to the basal ganglia network, the visual network, and the sensorimotor network in patients with asthma using functional magnetic resonance imaging (30). The current study found that the functional connectivity of the human brain changes dynamically over time (31). The dynamic functional connection analysis considers the time fluctuation of the functional connectivity in a short time range, and the time variability reflected by it is closely related to the adaptability and efficiency of the nervous system (32). Some evidence suggests that time variability can be used to explain the internal variability of subjects in disease (32, 33). These connection states with different time-varying characteristics may be of great value in evaluating the development of the patient's condition. The dynamic intrinsic brain activity (IBA) index is more sensitive than the static index to detect abnormal brain changes, and over time, the dynamic index is expected to become a novel, more powerful, and more sensitive neurobiological index related to imaging (34, 35). At present, dynamic functional connection (dFC) has successfully evaluated a variety of mental and neurological disorders. The study found that patients with Alzheimer's disease had abnormal dynamic functional connectivity in cognitively related brain regions and successfully predicted the degree of preclinical memory loss (36–38). Another study showed that abnormal dynamic connections of the brain network in patients with Parkinson's disease are not only related to cognitive dysfunction but also lead to motor disorders (39, 40). In addition, the changes in dynamic functional connections of the brain network can predict the effect of drug treatment for Parkinson's disease (41).

DFc is expected to reveal hypoxic brain dysfunction's internal variability and temporal dynamic changes, including asthma (31, 42). As one of the methods of the dFC analysis, dynamic voxel mirror homotopy connection (dVMHC) can provide a quantitative index of neural activity intensity that is more sensitive and specific than resting VMHC, which is vital to reveal the mechanism of abnormal brain function in asthma. Consequently, we investigated the changes in cerebral hemispheric dynamic functional connections in patients with asthma by analyzing the changes in dVMHC between cerebral hemispheres. By analyzing changes in dVMHC, we aimed to better understand the mechanisms underlying abnormal brain function in patients with asthma and guide clinical treatment.

## Materials and methods

### Participants

A total of 31 patients with BA were treated in Jiangxi Provincial People's Hospital (Nanchang City, Jiangxi Province), including 17 men and 14 women. Patients with asthma met the following criteria: (1) intermittent hypoxia and wheezing symptoms; (2) inhalation of bronchoconstrictor causes a 20% decrease in the first second forced expiratory volume (FEV1), or the use of bronchodilator FEV1 reversibly increases by more than 12% and more than 200ml); (3) there is no respiratory infection; and (4) there is no cardio-cerebrovascular disease.

We selected 31 healthy individuals (17 men and 14 women) whose background and basic identity information matched BA. All healthy participants met the following criteria: (A) no metal or other foreign bodies; (B) no MRI contraindications; (C) no organic lesions; and (D) no mood swings.

### MRI data acquisition

Data were collected using 3-Tesla MR scans with eight-channel head coils. The subjects were instructed to keep their eyes closed and remain awake throughout the scan, and their heads were fixed with a soft cotton pad to facilitate image collection. Earplugs were worn to reduce scanning noise. (1) The functional image of the resting state used an echo plane imaging sequence with the following parameters: echo time (TE) = 25.0 ms, repetition time (TR) = 2,000.0 ms, flip angle (FA) = 90°, field of view (FOV) = 220.0 × 220.0 mm, matrix = 64 × 64, voxel size = 3.6 × 3.6 × 3.6 mm. Thirty-five axial images were collected at each time point. (2) High-resolution T1W1 image: TE = 3.7 ms, TR = 8.9 ms, FA = 12°, field of view =240 × 240 mm, matrix = 256 × 256, Voxel size was the same as above.

### rs-fMRI data processing

We used DPABI (http://www.rfmri.org/dpabi) software to process the rs-fMRI data of all participants (43). Our main steps are as follows: (1) Eliminate the first 10 unstable time point signals from all participants in the initial stage of data acquisition; (2) make sure the acquisition time and head movement of different slices are accurate; (3) reduce the influence of image interference caused by noise, enhancing the statistical hypothesis's validity; (4) data from unrelated frequency bands were filtered to reduce noise; (5) controlling the interference of covariates using linear regression analysis; (6) continue the image registration for the brain tissue of different sizes and shapes, standardize these images with the standard template, and then resample the processed individual data to solve the mismatch between the scanning space and the obtained brain data.

### Dynamic VMHC analysis

The dynamic VMHC was calculated using the time-dynamic analysis (TDA) toolbox of DPABI. To detect and analyze the whole brain's dynamic VMHC variability, we used a sliding window analysis method (44, 45). To maximize the statistical efficiency of intra-window and cross-stage analyses, we used a sliding window length of 32tR (64s) and a shift step of 1tr (2s) (46). In these sliding windows, we calculated the VMHC of each voxel in the brain. We then Z-standardized these indicators using the gray matter masks of all voxels. The standard deviation (SD) of each standardized VMHC over the entire time window was calculated to evaluate the dVMHC.

### Statistical analysis

First, the SPSS 25.0 version was used to evaluate the clinical data between the case and healthy control groups. Second, the DynamicBC (47, 48) (www.restfmri.net/forum/DynamicBC) toolbox was used to analyze the dVMHC values of all subjects. Considering age, sex, and body mass index as covariates, the standardized dVMHC of the two groups was tested using a double-sample *t*-test with an SPM12 toolkit to evaluate the difference in dVMHC variability between the two groups. We also compared the multiple corrections using the Gaussian method [Gaussian random field (GRF) (two-tailed, voxel-level *P* < 0.01, GRF correction, and cluster-level *P* < 0.05)].

## Result

### Demographic and clinical characteristics

In this study, there was no significant difference in age, sex, education level, and body mass index between patients with bronchial asthma and healthy controls (*P* > 0.05) (Table 1).

**Table 1:** Comparison of general data between BA group and HC group.

**Characteristic**	**BA**	**HC**	**t**	***P*** **-value**
Men/women	17/14	17/14	N/A	0.708
Age (years)	51.52 ± 5.20	51.24 ± 5.17	0.258	0.794
Weight (kg)	63.46 ± 7.12	63.21 ± 6.58	0.196	0.844
BMI (Kg/m^2^)	22.6	21.7	0.15	0.821
Duration of asthma	27 ± 6.19	N/A	N/A	N/A

BA group, bronchial asthma group; HC group, healthy control group; *P* > 0.05.

### Differences between dynamic voxel mirror homotopy connection groups

**Figure 1:**
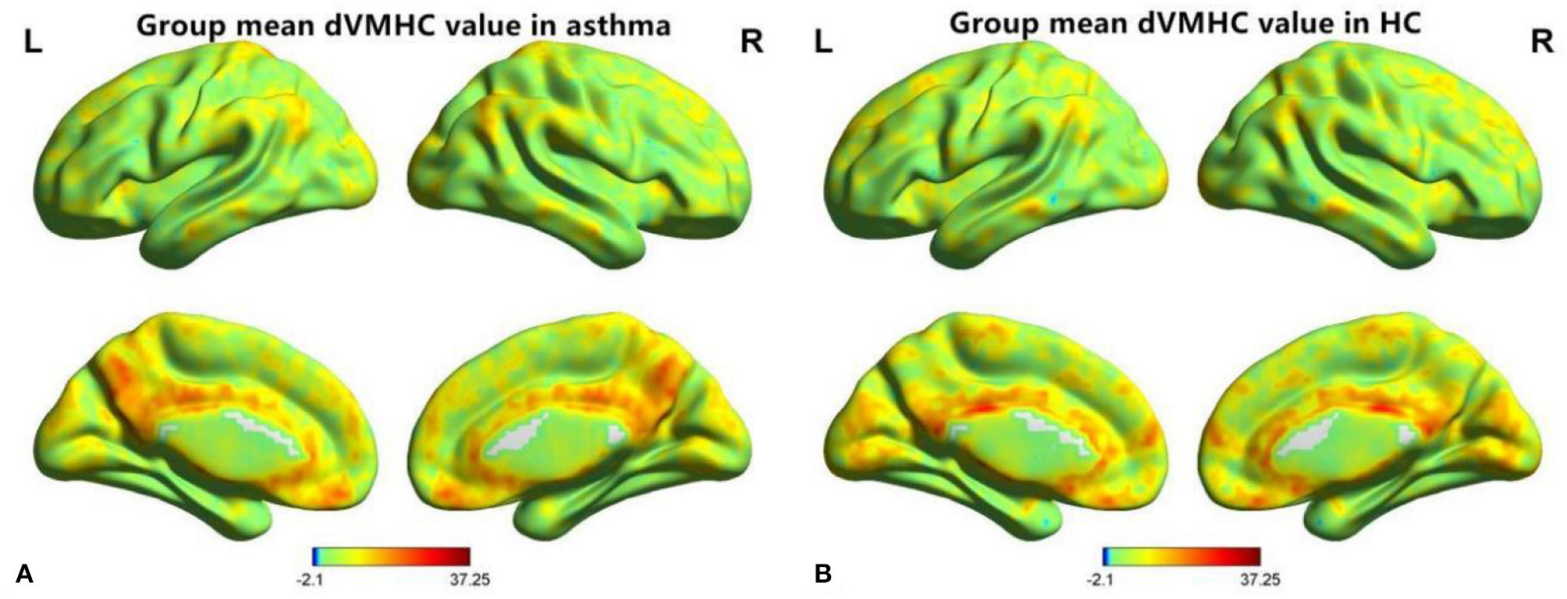
A diagram of dynamic voxel mirror homotopy connection distribution of resting functional MR imaging 0.01~0.08 Hz in the asthma group **(A)** and the healthy control group **(B)**, “L” is indicated on the left and “R” on the right.

**Figure 2:**
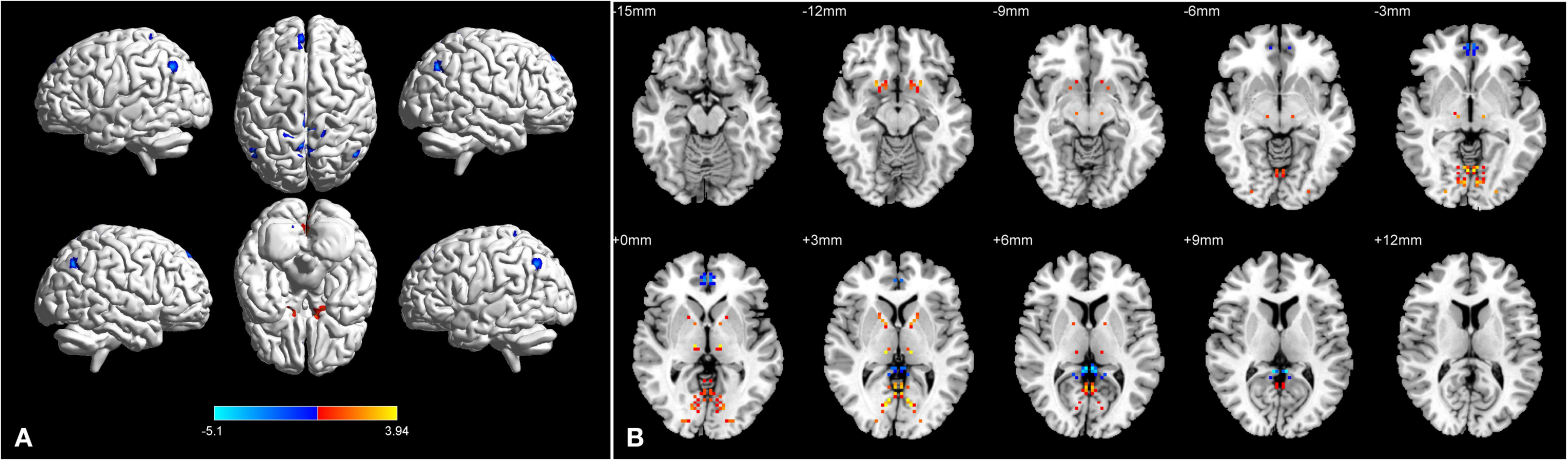
Brain regions with significant differences in dVMHC between BA group and HC group in 3D picture **(A)**. Significant differences in dVMHC values between the patients with asthma and HCs in 2D picture **(B)**.

The location and spatial distribution of the difference in dVMHC between the bronchial asthma case group and the healthy control group are shown in Table 2 and Figures 1, 2. The Gaussian random field specifies the standard (voxel-level *P* < 0.01, cluster level *P* < 0.01). After correction, the brain regions of LING and CAL, the medial superior frontal gyrus (medSFG), the cingulate gyrus (CG), and the supplementary motor area (SMA) in the asthma group were significantly different from those in the normal control group. Among them, dVMHC in the lingual gyrus (LING) and the calcarine sulcus (CAL) in the bronchial asthma group were higher than those in the healthy control group. In addition, our study observed a decrease in dVMHC in the medSFG CG, and SMA in the BA group.

**Table 2:** Differences in dVMHC values between the patients with asthma group and the healthy subjects group on fMRI.

**Cluster**	**Brain areas**	**Number of voxels**	**Peak MNI coordinate**			**Peak intensity**
			**x**	**y**	**z**	
1	Lingual_gyrus (LING)R/L	19	9	−69	3	3.7546
2	Superior frontal gyrus Medial_R(SFGmed)	12	3	45	0	−3.9125
3	Anterior Cingulate gyrus__ L(ACG)	12	−3	45	0	−3.9125
4	Lingual_gyrus (LING)R	14	3	−63	3	3.6948
5	Calcarine cortex L (CAL)	14	−3	−63	3	3.6948
6	Posterior Cingulate gyrus_R (PCG)	10	3	−39	6	−5.1017
7	Posterior Cingulate gyrus__L (PCG)	11	−3	−39	6	−5.1017
8	Middle Cingulate gyrus R(MCG)	83	3	−33	45	−4.4216
9	Middle Cingulate gyrus _L(MCG)	83	−3	−33	45	−4.4216
10	Supplementary_Motor_Area_R(SMA)	14	3	12	45	−3.5688
11	Supplementary_Motor_Area_L (SMA)	14	−3	12	45	−3.5688

Gaussian random field theory (double-tailed, voxel-level P < 0.01, clustering level P < 0.05), GRF correction, P < 0.01) was used for multiple comparisons. dVMHC is a dynamic mirror homotopy functional connection; BA, bronchial asthma; MNI, Montreal Institute of Neurology; X y z denote the MNI axis.

## Discussion

In the current study, we used the dVMHC values to analyze changes in brain function in different brain regions and found interesting results. Compared to the normal control group, the dynamic VMHC of the lingual gyrus (LING) and the calcarine sulcus (CAL) was significantly higher. Furthermore, in our research, we observed that the BA group's dVMHC in the medial superior frontal gyrus (medSFG), the cingulate gyrus (CG), and the supplementary motor area (SMA) was lower than that of the HC group.

DVMHC combines VMHC with the sliding window method, which can more effectively capture cerebral hemispheric activity's coordinated and dynamic characteristics. DVMHC focuses on reflecting the time stability of internal brain activity, while static VMHC is intended to reflect the intensity of internal brain activity. Although the lower time variability represents a relatively more stable functional state, we believe that this is due to the existence of the “decompensation mechanism,” which may improve the patient's memory to some extent, but cannot effectively replace the original function. It has been confirmed in some studies that low time variability means a decrease in cognitive flexibility (49, 50).

The calcarine sulcus lies in the medial occipital lobe, which divides the occipital lobe into the cuneate gyrus and the lingual gyrus (51). As advanced visual cortexes, CAL and LING are involved in integrating visual information and forming conscious perception (30, 52). Earlier studies in patients with asthma have confirmed that the function of the left calcarine is abnormal (53). In this study, we found that the dVMHC of the calcarine (BA19) and the lingual gyrus (BA18) increased. CAL and LING are all part of the visual network (VN) (54). The increase in time variability reflected by dVMHC indicates that the frequency of functional connection state transitions in the visual network in patients with BA has increased. This unstable conversion mode may affect the information transmission efficiency of the visual network and lead to abnormal function. In addition, the visual cortex is associated with attention bias, and people with asthma tend to show symptoms of depression, which usually makes their attention more likely to be affected by negative stimuli (55). This suggests that the increased variability of the visual network may reflect the attention bias caused by emotional disorders in patients with asthma (56). At the same time, Li et al. (20) also proved that the widespread decline of regional function in the visual network is the core change affected by asthma. Therefore, the abnormal variability of the visual network may be a specific feature of asthma, especially the key changes involved in emotional bias in asthma. In this study, we used the dVMHC method to more acutely detect the changes in visual function and the attention bias caused by emotional disorders in patients with BA.

The cingulate gyrus and the medial prefrontal lobe are the key areas of the default mode network (DMN). When the brain is idle to complete random thinking activities, DMN remains active, but when we focused on a certain difficult cognitive activity, it is suppressed. The DMN is an important network containing cognitive and emotional regulation systems (57, 58). Emotion and stress are thought to be triggers of bronchoconstriction. Several studies linked affective disorders with lung function deterioration or inflammation of the respiratory tract (59–63). An emotion induction experiment showed that 20–40% of patients developed emotion-related bronchoconstriction (64, 65). The researchers found that negative emotional stimulation reduced the activation of DMN-related areas, leading to more severe respiratory inflammation and worse asthma control (66). The cingulate gyrus belongs to the para-limbic system and is a key region of the DMN, which mainly controls emotion formation and cognitive activities. The cortical area of the cingulate gyrus is believed to be related to bronchospasm caused by emotional stress (67, 68). In addition, the study also found activation of the prefrontal lobe in patients with COPD (20, 69). The degree of respiratory distress was also closely associated with prefrontal cortex activation in patients with asthma (70). MPFC is activated by strong emotions (71). In addition, Dehdar et al. (72) found that the prefrontal lobe of patients with asthma is related to the regulation of anxiety. This suggests that there may be a relationship between the function of respiration and the functional changes of MPFC in different emotional states. Respiratory distress in patients with asthma may be explained by abnormal function of the cingulate gyrus and the prefrontal lobe. Treatments to improve emotional symptoms may therefore help control asthma symptoms. In addition, an earlier study found a positive correlation between inflammatory factors and functional changes in the superior frontal gyrus (59). The executive control network (ECN) also contains MPFC and CG (73, 74). ECN is related to emotional inhibition (73). The changes in dVMHC in these networks explain the dysfunction of emotion regulation in patients with BA.

Furthermore, ACC is also an important node of the highlighted network (SN) (75). SN can recognize the outer incentive that gains attention from a large quantity of sensory information. External stimulation can induce the insular lobe to generate ECN initiation signals and inhibit the resting activity of DMN. This network switching participates in advanced cognitive activities (75–77). DVMHC was significantly reduced in these brain networks in our study. Therefore, the changes in DVMHC in these brain networks can reflect airway hyperresponsiveness, inflammatory progression, and dyspnea in patients with asthma mediated by emotional regulation and cognitive dysfunction.

A sensorimotor network includes the precentral gyrus, the postcentral gyrus, the premotor area (PMA), and the supplementary motor area (SMA). SMN is responsible for monitoring our response to external stimuli. The SMA belongs to the motor system of the prefrontal cortex. It is located in the dorsomedial superior frontal gyrus, along with the posterior margin of the prefrontal sulcus and the primary motor cortex (PMC). The cingulate groove bounds the inside. However, there are no clear anatomical landmarks on the lateral and anterior sides. It mainly mediates information processing in cognitive function and can determine the specific sequence of motor sequences performed at lower levels of PMC. Functionally, it can be divided into the SMA front area and the SMA tail region. The front area is related to complex task processing, and the tail is related to body motion processing (78, 79). It is functionally divided into two parts: the snout part is involved primarily in cognitive activities during behavior changes, while the tail part is mainly related to the implementation of exercise plans (80–83). Studies found that thinning of the SMA cortex affects hemoglobin oxygen binding in people with chronic respiratory inflammation (84). Kojima et al. (85) found that the total hemoglobin of SMA increased under respiratory compensation in healthy people through cardiopulmonary exercise tests. Patients with BA with respiratory decompensation disorders may have abnormal hemoglobin levels in their SMA similar to patients with COPD. Therefore, we can infer that the lower functional connection flexibility of SMA is also caused by the decrease in oxygen saturation caused by asthma. As the key node of the sensorimotor network, SMA is related to the spontaneous activity and functional connection disorder of the SMN. In addition, the change in the sensorimotor network is related to the change in respiratory amplitude in patients with asthma (21). Persistent hypoxia caused by severe asthma will lead to a decrease in the SMN's ability to process somatosensory information about respiratory load (86). In our study, patients with BA had lower time variability in their connection between SMA and cingulate gyrus function. Therefore, functional changes in the SMN play a key role in predicting oxygen saturation and respiratory function in patients with BA. SMN represents a potential therapeutic target for asthma.

### Insufficient at present

First, the sample size is small, and more co-disease samples are needed to replicate and supplement our findings. Second, although MRI correction and data processing were performed in the resting state due to our technical limitations, there are still noise sources, including scanner drift, head movement, and physiological noise (such as breathing and heart rate), that greatly affect the dynamic analysis. A slight head movement or a brief deep breath will introduce strong signal fluctuations, which may manifest as a temporary change in the connection mode. Third, it is an open question on whether it is necessary to study the neural correlation frequency and appropriate time scale of connectivity changes. Fourth, patients with asthma take drugs, which may affect the reliability of our results. Fifth, correlation and coherence methods lack an appropriate model to solve the underlying structure of network interaction and cannot distinguish between the real variability in network interaction and the variability caused by random noise. These issues must be considered when interpreting the results reviewed below, and the development of modeling techniques for dynamic FC will be an important direction in the future.

## Data availability statement

The original contributions presented in the study are included in the article/supplementary material, further inquiries can be directed to the corresponding author.

## Ethics statement

The studies involving human participants were reviewed and approved by Jiangxi Provincial People's Hospital. Written informed consent was obtained from all participants before MRI scanning, and the research methods complied with the principles of the Declaration of Helsinki.

## Author contributions

TW, XH, and JW listed made a substantial, direct and intellectual contribution to the work, including, but not limited to, study design, execution, data acquisition, analysis, interpretation, drafting, revision, review, and approved its publication.
